# 1356. "Correlates of protection against SARS-CoV-2 post-vaccine infections in the Prospective Assessment of SARS-CoV-2 Seroconversion (PASS) Study"

**DOI:** 10.1093/ofid/ofad500.1193

**Published:** 2023-11-27

**Authors:** Emilie Goguet, Cara Olsen, William A Meyer, Sara Ansari, Tonia Conner, John H Powers, Si’Ana A Coggins, Wei Wang, Richard Wang, Luca Illinik, Margaret Sanchez Edwards, Belinda Jackson-Thompson, Monique Hollis-Perry, Gregory Wang, Yolanda Alcorta, Mimi Wong, David Saunders, Roshila Mohammed, Bolatito Balogun, Priscilla Kobi, Lakeesha Kosh, Kimberly A Bishop-Lilly, Regina Cer, Catherine Arnold, Logan Voegtly, Maren Fitzpatrick, Andrea Luquette, Francisco Malagon, Orlando Ortega, Edward Parmelee, Julian Davies, Alyssa R Lindrose, Hannah Haines-Hull, Matthew Moser, Emily C Samuels, Marana S Tso, Elizabeth Graydon, Allison M Malloy, David Tribble, Timothy Burgess, Wesley Campbell, Sara Robinson, Carol D Weiss, Robert O’Connell, Christopher C Broder, Simon Pollett, Eric D Laing, Edward Mitre

**Affiliations:** HJF, USUHS, Bethesda, Maryland; Uniformed Services University, Bethesda, Maryland; Quest Diagnostics, Baltimore, Maryland; Quest Diagnostics, Baltimore, Maryland; Uniformed Services University, Bethesda, Maryland; Support to National Institute of Allergy and Infectious Disease, Bethesda, MD; HJF/USUHS, Rockville, Maryland; US Food and Drug Administration, Silver Spring, Maryland; U.S. Food and Drug Administration, Silver Spring, Maryland; Henry M. Jackson Foundation for the Advancement of Military Medicine, Portsmouth, Virginia; Infectious Disease Clinical Research Program, Department of Preventive Medicine and Biostatistics, Uniformed Services University of the Health SciencesHenry M. Jackson Foundation for the Advancement of Military Medicine, Bethesda, Maryland; HJF, USUHS, Bethesda, Maryland; CTC, NMRC, Silver Spring, Maryland; Naval Medical Research Center, Bethesda, Maryland; CTC, NMRC, General Dynamics Information Technology, Bethesda, Maryland; CTC, NMRC, General Dynamics Information Technology, Bethesda, Maryland; Uniformed Services University of the Health Sciences, Bethesda, MD, USA, Bethesda, Maryland; HJF, TMU, USUHS, Bethesda, Maryland; HJF/IDCRP, USUHS, Bethesda, Maryland; HJF/IDCRP, USUHS, Bethesda, Maryland; HJF/IDCRP, USUHS, Bethesda, Maryland; Biological Defense Research Directorate, NMRC, Fort Detrick, Maryland; Biological Defense Research Directorate, NMRC, Fort Detrick, Maryland; Biological Defense Research Directorate, NMRC/Defense Threat Reduction Agency, Fort Detrick, Maryland; Biological Defense Research Directorate, NMRC/Leidos, Fort Detrick, Maryland; Biological Defense Research Directorate, NMRC/Leidos, Fort Detrick, Maryland; Biological Defense Research Directorate, NMRC/Leidos, Fort Detrick, Maryland; Biological Defense Research Directorate, NMRC/Leidos, Fort Detrick, Maryland; HJF, Infectious Diseases Clinical Research Program, Bethesda, Maryland; Infectious Disease Clinical Research Program, Department of Preventive Medicine and Biostatistics, Uniformed Services University of the Health Sciences, Bethesda, Maryland; Infectious Diseases Clinical Research Program, Henry M. Jackson Foundation, Bethesda, Maryland; HJF, USUHS, Bethesda, Maryland; HJF/USUHS, Rockville, Maryland; HJF/USUHS, Rockville, Maryland; HJF, USUHS, Bethesda, Maryland; HJF, USUHS, Bethesda, Maryland; HJF, USUHS, Bethesda, Maryland; Department of Pediatrics, Uniformed Services University of the Health Sciences, Bethesda, MD, USA, Bethesda, Maryland; Infectious Disease Clinical Research Program, Department of Preventive Medicine and Biostatistics, Uniformed Services University of the Health Sciences, Bethesda, MD, USA, Bethesda, Maryland; Infectious Disease Clinical Research Program, Department of Preventive Medicine and Biostatistics, Uniformed Services University of the Health Sciences, Bethesda, MD, USA, Bethesda, Maryland; Division of Infectious Diseases, Walter Reed National Military Medical Center, Bethesda, Maryland; Walter Reed National Military Medical Center, Bethesda, MD, Bethesda, Maryland; U.S. Food and Drug Administration, Silver Spring, Maryland; Infectious Disease Clinical Research Program, USUHS, Bethesda, Maryland; Department of Microbiology and Immunology, Uniformed Services University, Bethesda, MD, Bethesda, Maryland; Infectious Disease Clinical Research Program, Department of Preventive Medicine and Biostatistics, Uniformed Services University of the Health Sciences, Bethesda, MD, USA, Bethesda, Maryland; Department of Microbiology and Immunology, Uniformed Services University, Bethesda, MD, Bethesda, Maryland; Uniformed Services University, Bethesda, Maryland

## Abstract

**Background:**

We sought to determine pre-infection correlates of protection against SARS-CoV-2 post-vaccine infections.

**Methods:**

Serum and saliva samples from 176 BNT162b2-vaccinated adults in the Prospective Assessment of SARS-CoV-2 Seroconversion study were collected between October and December 2021 and assessed for serum and saliva levels of ancestral (WT) SARS-CoV-2 Spike (S)-specific IgG and IgA binding antibodies (bAb), and serum levels of Omicron BA.1 subvariant S-specific IgG bAb using a multiplex microsphere-based immunoassay. Serum samples were also assessed for WT S-specific bAb by two commercial assays, and neutralization activity against D614G, Delta (617.2), and Omicron BA.1 and BA.1.1 subvariants using a lentiviral pseudovirus neutralization assay. After the fall 2021 visit, participants reported all positive PCR and/or antigen tests for SARS-CoV-2. Duration, severity, and type of symptoms, as well as risk exposures and adherence to precautionary measures, were assessed by questionnaires during the spring 2022 visit.

**Results:**

Thirty-two participants (18.2%) developed post-vaccination infections (PVI) between December 7, 2021 and April 1, 2022. Pre-infection WT and BA.1 anti-S IgG bAb levels measured by MMIA and neutralizing titers (NT) against BA.1 and BA.1.1 were significantly higher in individuals that did not develop PVIs. WT anti-S IgG bAb levels greater than 5,000 binding antibody units/ml were associated with substantial protection against symptomatic PVI.

**Conclusion:**

Anti-S IgG bAb levels (directed against either WT or BA.1) and NT IgG titers both serve as correlates of protection against symptomatic PVI, with high levels associated with both protection against symptomatic infection and reduced severity of disease in those who develop PVI. Results of this study also suggest that commercial assays for anti-S bAb may need to be reformatted to enable detection of higher maximum values for use as predictors of increased susceptibility to SARS-CoV-2 infection.

**Disclosures:**

**David Tribble, MD, DrPH**, AstraZeneca: The IDCRP and HJF were funded to conduct an unrelated phase III COVID-19 monoclonal antibody immunoprophylaxis trial as part of US Govt COVID response **Timothy Burgess, MD, MPH**, AstraZeneca: The IDCRP and the Henry M. Jackson Foundation (HJF) were funded to conduct an unrelated phase III COVID-19 monoclonal antibody immunoprophylaxis trial **Simon Pollett, MBBS**, AstraZeneca: The IDCRP and the Henry M. Jackson Foundation (HJF) were funded to conduct an unrelated phase III COVID-19 monoclonal antibody immunoprophylaxis trial

